# The role of D-serine and glycine as co-agonists of NMDA receptors in motor neuron degeneration and amyotrophic lateral sclerosis (ALS)

**DOI:** 10.3389/fnsyn.2014.00010

**Published:** 2014-04-16

**Authors:** Praveen Paul, Jackie de Belleroche

**Affiliations:** Neurogenetics Group, Division of Brain Sciences, Department of Medicine, Imperial College LondonLondon, UK

**Keywords:** amyotrophic lateral sclerosis (ALS), motor neuron disease, D-serine, D-amino acid oxidase (DAO), neurodegeneration, N-methyl-D-aspartic acid receptor (NMDAR), apoptosis, autophagy

## Abstract

The fundamental role of D-serine as a co-agonist at the N-methyl-D-aspartate receptor (NMDAR), mediating both physiological actions of glutamate in long term potentiation and nociception and also pathological effects mediated by excitotoxicty, are well-established. More recently, a direct link to a chronic neurodegenerative disease, amyotrophic lateral sclerosis/motor neuron disease (ALS) has been suggested by findings that D-serine levels are elevated in sporadic ALS and the G93A SOD1 model of ALS (Sasabe et al., 2007, 2012) and that a pathogenic mutation (R199W) in the enzyme that degrades D-serine, D-amino acid oxidase (DAO), co-segregates with disease in familial ALS (Mitchell et al., 2010). Moreover, D-serine, its biosynthetic enzyme, serine racemase (SR) and DAO are abundant in human spinal cord and severely depleted in ALS. Using cell culture models, we have defined the effects of R199W-DAO, and shown that it activates autophagy, leads to the formation of ubiquitinated aggregates and promotes apoptosis, all of which processes are attenuated by a D-serine/glycine site NMDAR antagonist. These studies provide considerable insight into the crosstalk between neurons and glia and also into potential therapeutic approaches for ALS.

## Identification of a mutation in D-amino acid oxidase (DAO) in familial ALS

It has only recently been recognized that D-amino acids play an important physiological role in the central nervous system. The evidence is most convincingly demonstrated for D-serine through its action as a co-agonist at the N-methyl-D-aspartate receptor (NMDAR), a major glutamate receptor subtype, involved in synaptic plasticity (Mothet et al., 2000). An essential role for D-serine has been demonstrated in long-term potentiation (Yang et al., 2003), fear conditioning (Li et al., 2013) and nociception (Wake et al., 2001). Abnormal levels of D-amino acid have been reported in aging and Alzheimer's disease but it is only more recently that, a direct link has emerged between D-serine and a chronic neurodegenerative disease, amyotrophic lateral sclerosis (ALS). ALS is a common adult-onset neuromuscular disease characterized by selective loss of motor neurons leading to fatal paralysis with a life-time risk of 1 in 500. At the cellular level, the hallmark of ALS is the presence of TDP-43-positive ubiquitinated protein aggregates (Neumann et al., 2006).

The link between D-amino acids and disease was first indicated by findings that D-serine levels are elevated in sporadic ALS and also in the G93A SOD1 mouse model of ALS (Sasabe et al., 2007, 2012). Our own studies have identified a pathogenic mutation (R199W) in the enzyme that degrades D-serine, D-amino acid oxidase (DAO) and co-segregates with disease across multiple generations in a kindred with familial ALS (Mitchell et al., 2010). The familial form of ALS constitutes 10% of all cases of ALS and to-date the causal genes in over 70% of these families have been identified, the most common being *C9orf72*, a gene of unknown function, Cu/Zn-dependent superoxide dismutase (*SOD1*), TAR DNA binding protein 43 (*TARDBP*) and fused in sarcoma (*FUS*). Arginine^199^ is highly conserved across species from bacteria to man and lies within the active site close to the FAD and substrate binding sites of the enzyme. The effect of the R199W-DAO mutation is very profound abolishing enzyme activity (Mitchell et al., 2010). Furthermore, R199W-DAO, but not wild-type DAO, has marked toxic effects in motor neurons promoting apoptosis following transfection of primary motor neuron cultures and causing protein aggregate formation in motor neuron cultures (Mitchell et al., 2010). To further understand the involvement of D-amino acids and DAO in ALS, we used a cohort of sporadic ALS cases and controls (Anagnostou et al., 2010) to characterize their distribution in spinal cord and investigated the pathogenic effects of the FALS-associated mutation in cell culture.

## D-serine and DAO distribution in spinal cord

The distribution of DAO, D-serine and serine racemase (SR), the major biosynthetic for D-serine, in mammalian brain has been characterized extensively (Moreno et al., 1999; Williams et al., 2006; Verrall et al., 2007; Balu et al., 2014). DAO has a selective distribution, being particularly abundant in cranial motor nuclei such as facial nerve nucleus, magnocellular cells of the reticular formation, deep cerebellar nuclei and is present in cerebellum in both Purkinje cells and Bergmann glial cells (Mitchell et al., 2010; Paul and de Belleroche, 2012). Lower levels of DAO are generally found in forebrain compared to hind brain.

In human spinal cord DAO and D-serine immunoreactivity is prominent in motor neurons (Paul et al., 2014). DAO immunoreactivity is highly concentrated in spinal cord motor neurons, fibers and glial cells and at the cellular level, the distinct peroxisomal localization of DAO is evident (Paul et al., 2014). D-serine appears punctate and in vesicle-like structures throughout the cytosol of motor neurons and glial cells. SR is also present in both motor neuron and glial cells in control spinal cord. In ALS, levels of D-serine, DAO, and SR in spinal cord are all severely depleted reflecting the substantial loss of motor neurons with disease. Whilst SR-positive motor neurons loss occurs in ALS, SR-positive glial cells are substantially increased in ALS (Paul et al., 2014).

## Effect of mutant-DAO expression *in vivo*

A naturally occurring point mutation in DAO (G181R) has been identified in a mouse line, which shows abnormal locomotor activity (Almond et al., 2006) and like R199W-DAO has impaired DAO activity. Recently, this line (ddY/DAO^−^mice) was backcrossed with C57BL/6J mice and maintained as homozygotes (DAO^−/−^) in order to study the motor phenotype in more detail (Sasabe et al., 2012). Motor phenotype was markedly affected in these G181R-DAO mice. At 8 months abnormal reflexes were detected, characterized by retraction of hind limbs, as seen in the G93A SOD1 mouse model of motor neuron disease, which was accompanied by a reduction in motor neuron number of 24% in DAO^−/−^ mice. At 15 months, axonal degeneration was evident with muscle atrophy and increased ubiquitination was also detected at this time point. The study also showed that D-serine accumulated in the spinal cord during disease progression in the G93A SOD1 mouse model (Sasabe et al., 2012).

## Pathogenic mechanisms in cell culture

### Apoptosis

In order to dissect out the mechanisms of R199W-DAO-mediated toxicity, we studied effects occurring both in motor neurons and astrocytes, using a motor neuron cell line, NSC-34, and the C6 glioma cell line (Paul et al., 2014). Cells were cultured separately and in co-culture in order to characterize the different cellular actions of the mutant protein and also the interaction between neuronal and glial cells. Our earlier studies in primary cultures showed that not only did direct transduction of motor neurons with R199W-DAO cause apoptosis but also co-culture of glial cells expressing R199W-DAO with motor neurons lacking R199W-DAO had a similar effect on the viability of neuronal cells (Mitchell et al., 2010).

**Figure 1:**
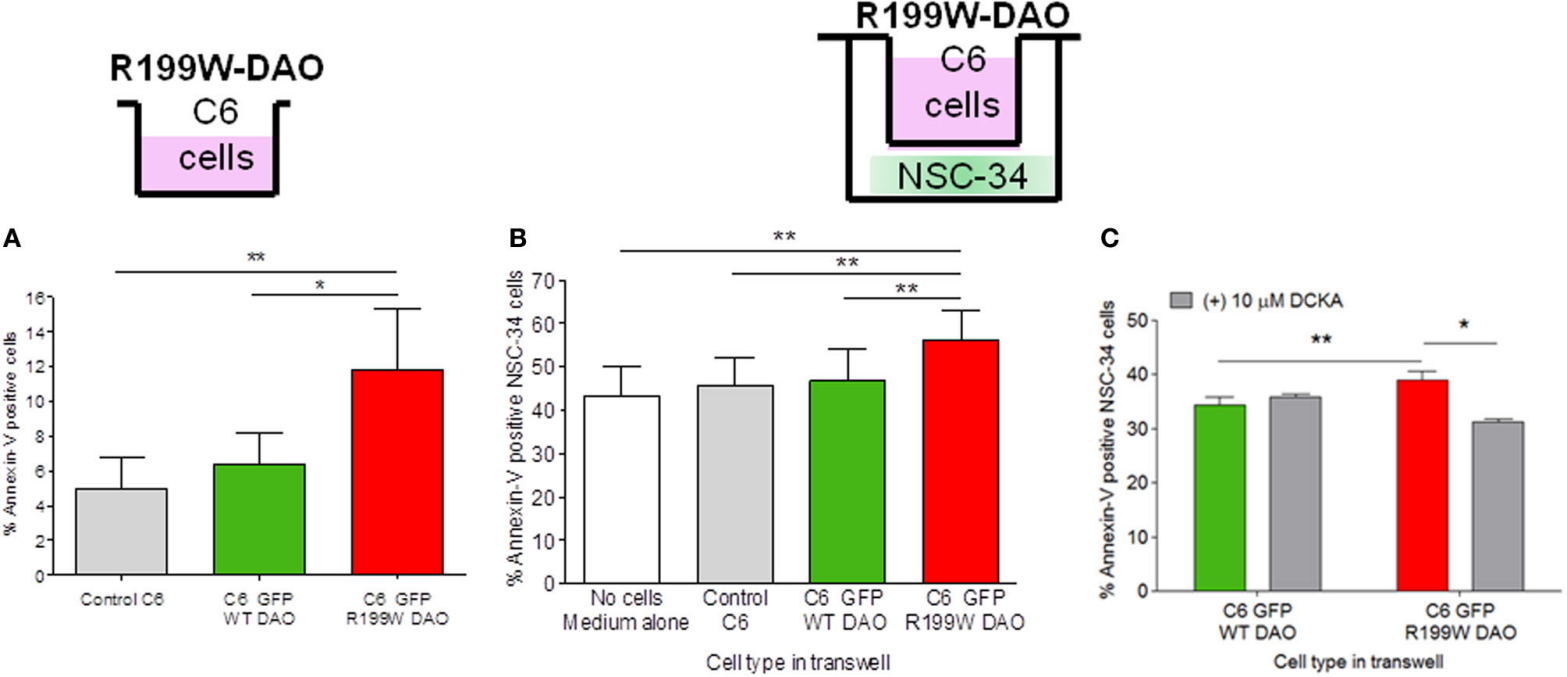
**R199W DAO promotes apoptosis in glial cells and neighboring neuronal cells**. Annexin V levels in **(A)** C6 glial cells permanently expressing WT or R199W DAO, **(B)** NSC-34 neuronal cells co-cultured with C6 glial cells, **(C)** NSC-34 cells treated with DCKA co-cultured with C6 cells and immunoblotted and quantified. Significant One-Way ANOVA subject to *post-hoc* testing with Bonferroni correction **(A,B)**. Paired *t*-test used in **(C)**. Values are means ± s.e.m., for *P*-values shown, ^*^P < 0.05, ^**^P < 0.01. *n* = 5, except **(C)** where *n* = 3. It should be noted that levels of annexin V in adherent cells are higher than those obtained in cell suspensions due to some activation caused by mechanical cell detachment. Data is taken from Paul et al. (2014).

C6 cell lines stably expressing wild-type or R199W-DAO were established and shown to exhibit low levels of apoptosis in culture (Figure 1A; Paul et al., 2014). For co-culture studies, these cells were cultured on a membrane insert suspended above differentiated NSC-34 cells using a transwell system. Although physically separated, the two cell lines shared the same medium. We co-cultured DAO (wild-type or R199W-DAO) expressing glial cells with NSC-34 cells and after 48 h in co-culture, NSC-34 cells were stained for annexin-V (an early apoptotic marker) and 7-AAD (a late apoptotic marker) and then assayed using flow cytometry. Co-culture of NSC-34 cells with R199W-DAO expressing glial cells increased annexin-V staining compared to co-culture with wild-type-DAO expressing glial cells (Figure 1B; Paul et al., 2014).

These results suggested that dysfunction of D-serine metabolism caused by a mutant allele leads to the release of a diffusible agent from glial cells that affects the viability of NSC-34 cells. One possible candidate is extracellular D-serine, a co-agonist at the NMDAR, which facilitates excitatory activity at this receptor. The source of D-serine may be activity-dependent release from either neuronal or astrocytic cells (Mothet et al., 2005; Kartvelishvily et al., 2006; Panatier et al., 2006; Martineau et al., 2013) and furthermore may involve amino acid transporters (Shao et al., 2009) on neurons that mediate release of D-serine and glycine that subsequently modulates NMDAR activity (Rosenberg et al., 2013). This was confirmed using 5,7-dichloro-4-hydroxyquinoline-2-carboxylic acid (DCKA), a selective antagonist at the glycine/D-serine binding site of the NMDA receptor which effectively prevents cell death due to NMDA or simulated ischemia in brain slices (Katsuki et al., 2004). We found that the increased apoptosis in NSC-34 cells induced by R199W DAO expressing glial cells was significantly attenuated by DCKA (Figure 1C; Paul et al., 2014). These results provide substantial evidence for a non-cell autonomous effect mediated by glia (Ilieva et al., 2009) that is similar to that reported when glial cells from G93A-SOD1 transgenic mice are co-cultured with motor neurons (Nagai et al., 2007) and indicates that glial-motor neuron interactions play an important role in ALS pathogenesis.

### Autophagy

**Figure 2:**
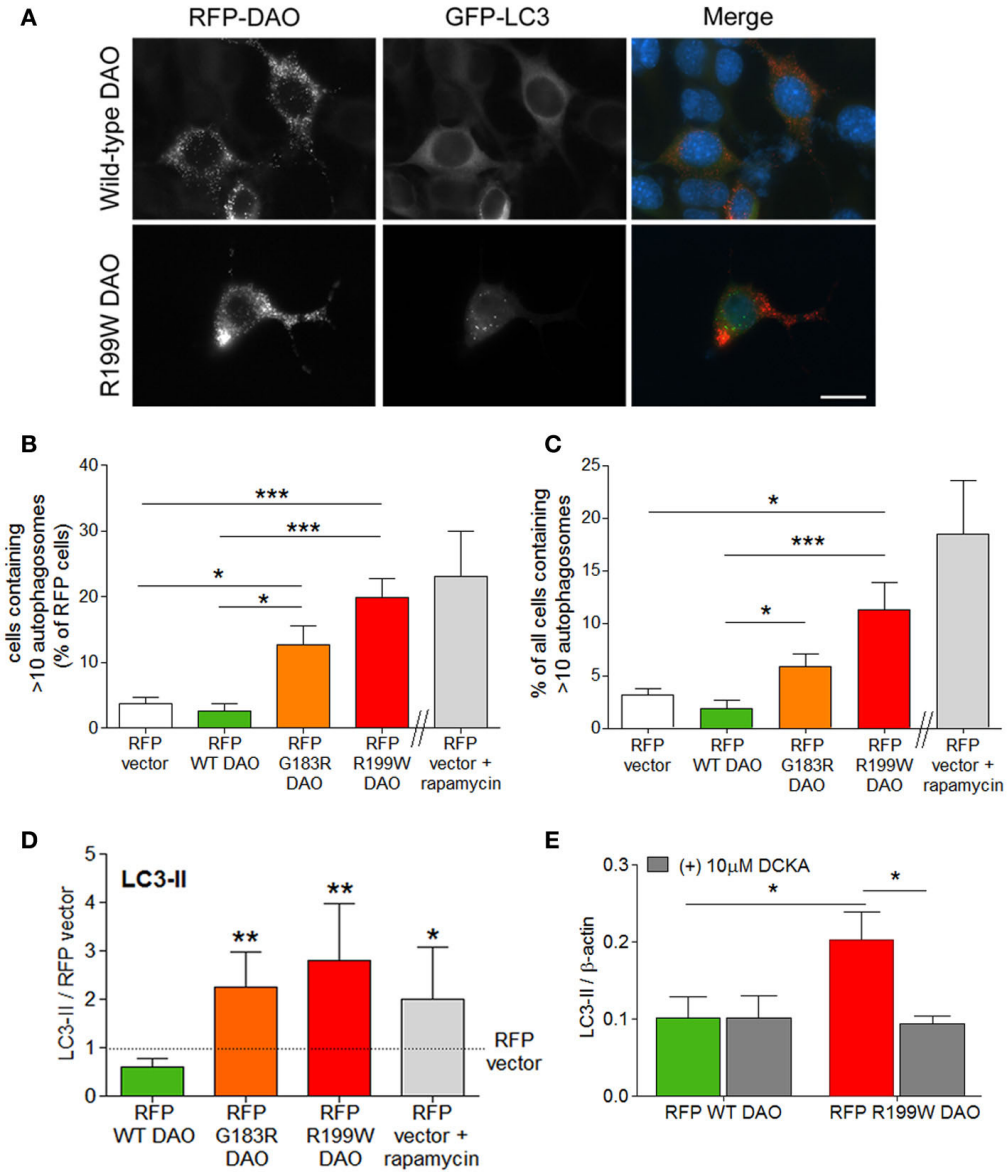
**R199W DAO promotes autophagy in neuronal cells**. NSC-34 cells were co-transfected with RFP-tagged DAO and GFP-tagged LC3. **(A)** Representative immunofluorescence images show RFP-DAO (red) GFP-LC3 (green) and DAPI nuclear stain (blue). Scale bar: 20 um. Number of **(B)** vector or DAO transfected cells or **(C)** cells containing >10 GFP-LC3 puncta or autophagosomes. Rapamycin induced autophagy was used as a positive control. **(D)** Levels of LC3-II were calculated using densitometry and normalized to protein levels of RFP-vector. **(E)** NSC-34 cells were treated with DCKA, immunoblotted and quantified. Significant One-Way ANOVA with Friedman's test subject to *post-hoc* testing with Dunn's multiple comparison test **(B–D)** or Two-Way ANOVA **(E)**. Values are means ± s.e.m., for *P*-values shown, ^*^P < 0.05; ^**^P < 0.01; ^***^P < 0.001. *n* = 4, except **(B,C)** where *n* = 6. Data is taken from Paul et al. (2014).

Since R199W-DAO expressing motor neurons are characterized by ubiquitinated protein aggregates (Mitchell et al., 2010), we measured the effects of R199W DAO on the major pathways involved protein degradation, the ubiquitin-proteasome system (UPS) and autophagy, both of which were triggered by R199W-DAO. The most substantial flux was seen in autophagy, with large increases occurring in the generation of autophagosomes associated with increased levels of the microtubule associated protein light chain 3-II (LC3-II) protein, a marker/mediator of autophagosome formation. Conversion of LC3-I to its lipidated form LC3-II denotes an induction of autophagy associated with an increase in autophagosomes (Kabeya et al., 2000). Following the co-transfection of NSC-34 cells with GFP-tagged LC3 and RFP-tagged DAO, a five-fold increase in the number of cells containing >10 GFP-tagged LC3 puncta/autophagosomes was evident in cells expressing RFP-tagged R199W DAO compared to wild-type or vector transfected cells (Figures 2A–C; Paul et al., 2014).

Increased LC3-positive autophagosome production also occurred in cells expressing G183R DAO, the human equivalent of a spontaneously occurring mutation in mice, which abolishes enzyme activity and leads to enhanced NMDA receptor activity *in vivo* (Almond et al., 2006). Levels of LC3-II protein were quantified by Western blot analysis and showed a similar magnitude of effect for both DAO mutations (Figure 2D; Paul et al., 2014). Increased LC3-II levels are also observed in spinal cord motor neurons of the SOD1 (G93A) mouse model of ALS (Zhang et al., 2011) and in ALS motor neurons (Sasaki, 2011). To determine whether the increased autophagic flux was linked to co-agonist regulation of NMDAR activity, we tested the effect of DCKA on LC3-II induction. DCKA significantly reduced the increase in LC3-II levels in R199W-DAO expressing cells but had no effect in WT-DAO expressing cells (Figure 2E; Paul et al., 2014) strongly suggesting that the increased autophagy caused by R199W-DAO was triggered via the NMDAR (Paul et al., 2014). A similar example of NMDAR activation leading to an increase in LC3-II and autophagic-mediated cell death has previously been demonstrated in cerebellar granule cells (Sadasivan et al., 2010). These findings further substantiate the potential contribution of the NMDAR in triggering the pathogenic effects of mutant DAO.

## Is there a role for glycine, a second co-agonist at the NMDA receptor in ALS pathogenesis? CSF and plasma glycine levels are elevated in ALS

The findings both in ALS spinal cord and SOD1 mice that D-serine is elevated compared to controls provide a potential link between D-serine and ALS pathogenesis. However autopsy cases generally represent end-stage conditions where extensive motor neuron loss has already occurred and extensive degeneration is in progress, which obscures early events. Previous studies in our laboratory examining levels of common amino acids in CSF in ALS showed significant increases in four amino acids (L-threonine, glycine, L-valine and L-phenylalanine) compared to controls, the greatest change being an increase of 57% in glycine which suggested a potential impairment in glycine transport (de Belleroche et al., 1984). In a subsequent follow-up study, we showed that glycine clearance from plasma after a glycine challenge was significantly impaired in classical ALS (*n* = 23) and in two overlapping ALS-like conditions, progressive bulbar palsy and primary lateral sclerosis (total ALS, *n* = 32), compared to normal controls and a control neurological group consisting of conditions associated with similar muscle loss (total controls, *n* = 26) (Lane et al., 1993). The most marked difference between ALS and controls was a greater than 2-fold increase in CSF levels of glycine at 2.5 h, at which time glycine levels have reached a maximum.

An impaired clearance of both glycine, as indicated from these results (Lane et al., 1993), and also of D-serine as indicated from the studies of (Sasabe et al., 2007, 2012) in ALS, could both lead to a potentiation of NMDA receptor currents. The balance between these co-agonists is likely to be complex and selectively modulated by multiple factors. However, an understanding of the mechanisms regulating the selective recruitment and properties of either glycine or D-serine in the modulation of NMDAR activity is now emerging and shows both a functional specificity for each co-agonist and interdependence that further shows regional variation.

In hippocampus and amygdala (Li et al., 2009, 2013), D-serine is thought to serve as the endogenous agonist responsible for tonic activation mediating NMDAR-mediated currents (EPSCs) in response to low levels of synaptic activity, which effect is diminished by DAO but not glycine oxidase (GO), and is reduced in serine racemase knock-out mice SR^−/−^, where D-serine synthesis is reduced. On the other hand, glycine facilitates NMDAR-mediated currents after afferent stimulation, diminished by GO but not by DAO (Li et al., 2013). Under these conditions, activity-dependent release of glycine from astrocytes is sufficient to support synaptic activation. Thus, both co-agonists are likely to play a role in the induction of LTP, as depletion of D-serine by DAO or SR^−/−^ knockdown or depletion of glycine by GO reduce LTP (Li et al., 2013).

The interplay between glycine site co-agonists has not been extensively characterized in brain stem and spinal cord. However, studies in rat hypoglossal motor neurons suggest that an increase in synaptically released glycine in response to metabolic stress may play a role in exacerbating NMDA receptor-mediated excitotoxicity in motor neurons (Kono et al., 2007). Regardless of the co-agonist, a selective antagonist for the glycine site on the NMDAR may have therapeutic potential in reducing excitotoxicity, as evidenced from the use of DCKA in the cell culture studies described above.

## A generic model of neurodegeneration

Experimental evidence obtained to date highlights the selective localization of DAO in spinal cord motor neurons, brain stem motor nuclei, cerebellum and other CNS centers involved in motor control. DAO is also found in glial cells in spinal cord and cerebellum. This distribution of enzyme activity is consistent with the lower levels of D-serine found in posterior brain compared to forebrain. The primary findings that implicate D-serine in ALS and potentially in neurodegeneration in general are that elevated levels of D-serine in spinal cord are found in ALS and the SOD1 mouse model of ALS (Sasabe et al., 2007, 2012). This suggests that elevated levels of D-serine contribute to motor neuron degeneration which is supported by the findings concerning two mutations in DAO that virtually abolish enzyme activity, one that is associated in familial ALS in man and a second found in the mouse that causes a motor phenotype similar to that seen in the SOD1 mouse model of ALS (Sasabe et al., 2012). In contrast, SR deletion that reduces D-serine levels protects against cerebral ischemia and excitotoxicity (Mustafa et al., 2010).

In order to further substantiate this concept, we have explored the potential toxic properties of the FALS-associated R199W-DAO mutation in cell culture and have shown that whether expressed in astrocytes or motor neurons the mutation promotes apoptosis but most interesting when expressed in glial cells the mutant protein is able to initiate apoptosis in neighboring neurons in co-culture that lack the mutation. The way in which this effect is transmitted between cells appears to be crucially dependent on the presence of an NMDAR co-agonist as the effect is blocked by the selective antagonist DCKA. In the situation where DAO is inhibited due to the presence of a mutation, D-serine levels are elevated (Sasabe et al., 2007) and could potentially promote NMDAR-mediated excitotoxic cell death. Furthermore, D-serine has been shown to increase the affinity of glutamate binding to the NMDAR even in the absence of changes in glutamate levels (Crow et al., 2012).

It is of considerable importance to note that increased synthesis of D-serine by neuronal and glial SR is known to be activated by cellular stressors such as AMPA, amyloid-beta and lipopolysaccharide (Wu and Barger, 2004: Wu et al., 2007; Paul et al., 2014) which may lead to the elevated levels of D-serine found in sporadic cases of ALS. These and other factors such as oxidative stress and ageing may further contribute to the etiology of sporadic ALS and other neurodegenerative conditions. Most importantly, future studies are needed to determine the spatial distribution of D-serine and glycine uptake mechanisms and the different populations of NMDARs in the spinal cord that may be gated by different co-agonists.

### Conflict of interest statement

The authors declare that the research was conducted in the absence of any commercial or financial relationships that could be construed as a potential conflict of interest.
